# *FOXD3* Suppresses the Proliferation of CRC Bone Metastatic Cells *via* the Ras/Raf/MEK/ERK Signaling Pathway

**DOI:** 10.2174/1386207326666230505111008

**Published:** 2024-02-09

**Authors:** Kangwei Wang, Yan Chu, Hongqiang Zhang, Xinglong Qu, Bing Wang, Yu Han

**Affiliations:** 1 Department of Oncological Surgery, Minhang Branch, Cancer Hospital, Fudan University, Shanghai, China;; 2 Shanghai Fifth People's Hospital, Fudan University, Shanghai, China

**Keywords:** *FOXD3*, CRC, bone metastasis, EMT, TCGA, EGFR/ERK signaling pathway

## Abstract

**Background:**

The improvements in the treatment of colorectal cancer (CRC) and prolongation of survival time have improved the incidence of bone metastasis. Forkhead box D3 (FOXD3) is involved in the development of CRC. However, the role and mechanism of FOXD3 in CRC bone metastases development are unknown.

**Objective:**

Using the combined bioinformatics and cytology experimental analyses, this study aimed to explore the mechanistic role of FOXD3 in the bone metastasis of colon cancer, thereby aiding in the treatment of colon cancer bone metastasis and identification of drug-targeting markers.

**Methods:**

First, the changes in the expression levels of the *FOXD3* gene and differentially expressed genes (DEGs) between the colon cancer samples and colon cancer metastases were obtained from The Cancer Genome Atlas (TCGA) database. Then, the correlations of the *FOXD3* gene with the DEGs were identified. Next, the effects of the FOXD3 on the proliferation and invasion abilities of colon cancer bone metastatic cells were identified using Cell Counting Kit-8 (CCK8) and Transwell cell migration assays, respectively. In addition, Western blot analysis was used to identify the expression levels of the proteins related to the EGFR/Ras/Raf/MEK/ERK (EGFR/ERK) signaling pathway and epithelial-to-mesenchymal transition (EMT).

**Results:**

*FOXD3* was downregulated in colon cancer and could interact with multiple DEGs in colon cancer bone metastases. *FOXD3* gene knockdown could increase the proliferation of human colon cancer bone metastatic cells and their invasive ability. *FOXD3* gene knockdown could activate the expression of EGFR/ERK signaling pathway-related proteins and inhibit/promote the expression of EMT-related proteins, which in turn promoted the proliferation and metastasis of LoVo cells from colon cancer bone metastases.

**Conclusion:**

Overall, this study demonstrated that the downregulation of the FOXD3 gene might promote the proliferation of colon cancer bone metastatic cell lines through the EGFR/ERK pathway and promote their migration through EMT, thereby serving as a promising therapeutic target.

## 1 INTRODUCTION

Colon cancer, also known as colorectal cancer (CRC), is the most common gastrointestinal tract cancer. CRC is ranked second and third among the most common cause of cancer-related deaths in the United States [1] and worldwide [2, 3]. Despite the improvements in CRC treatment and prolonging survival time, the incidence of bone metastasis was reported to be 3.7-11% [4]. Wang *et al.* analyzed the clinical data of 26,170 CAC patients and reported almost 7% incidence of bone metastasis [5]. Usually, there are multiple CRC bone metastases, and the local bone metastases are more common in the spine and pelvis, especially in the lumbar spine, sacrum, and thoracic spine, and rare in the ribs, limb bones, and skull [6]. Bone metastases do not respond to radiotherapies and chemotherapies. Therefore, their treatment and prevention are difficult, and the metastatic tumors destroying the bone tissues, resulting in pain and pathological fractures in bone, thereby seriously affecting the quality of patients’ lives and survival rates. Considering the genetic diversity of organisms, the insensitivities of the most malignant tumor patients to chemoradiotherapies might be due to the regulatory genes or signal regulatory networks related to chemoradiotherapy resistance [7]. Therefore, discovering new anti-cancer targets and exploring their underlying mechanisms in malignant tumors might help in choosing the optimized clinical treatment method for the patients, improve their postoperative survival rates, and enhance their quality of life.

The human forkhead box (*FOX*) gene family is a class of transcription factors, having 50 members in the human genome, which are divided into 19 subfamilies [7, 8]. The *FOX* gene family plays a complex and significant role in human growth and development. The abnormal expression of these genes might lead to congenital diseases, diabetes, or carcinogenesis. The attenuated or enhanced expression of *FOX* family genes causes cellular variation and promotes tumorigenesis and cancer progression [9]. *FOXD3* is located on the human chromosome 1p31 and is an important member of the *FOX* gene family. It can combine with the transcriptional repressor Grg4 and inhibit the transcription of genes. Recent studies showed that the *FOXD3* gene could transcriptionally interact with other genes and participate in different biological processes, such as cell differentiation, metabolism, proliferation, migration, apoptosis, and tumorigenesis. *FOXD3* gene could act as a tumor suppressor gene in a variety of tumors, including malignant melanoma, lung cancer, colorectal cancer, liver cancer, breast cancer, and thyroid cancer [10-16]. However, there are no studies on the effects of the *FOXD3* gene on the bone metastases of colon cancer.

Therefore, this study aimed to evaluate the downregulatory effects of the *FOXD3* gene on the proliferation and death of bone metastatic cells in human colon cancer. *In-vitro* experiments were performed to explore the molecular role of the *FOXD3* gene in the bone metastasis of colon cancer.

## 2 MATERIALS AND METHODS

### 2.1 Bioinformatics Analysis

The *FOXD3* gene expression profiles were downloaded from TCGA (https://portal.gdc.cancer.gov/) and the University of Alabama at Birmingham Cancer Data Analysis Portal (UALCAN) (http://ualcan.path.uab.edu/index.html) databases. In the UALCAN database, the term ‘FOXD3’ was searched in the ‘Gene’ field and ‘COAD’ was selected in the ‘Datasets Selection’ field. The R packages TCGAbiolinks and bio mart were used to download the data from the TCGA database and convert the Ensembl gene IDs to gene symbols, respectively. The DESeq2 package was used for the differential analysis. The threshold of Log fold-change (logFC) was set to 0.5 and the expression profiles of the *FOXD3* gene and differentially expressed genes (DEGs) were extracted. Pearson correlation analysis was used for the gene correlation analysis. The correlated genes were screened with a *P*-value of <0.05 and an absolute value of correlation (cor) greater than 0.3.

### 2.2 Cell Culture

The human colon cancer bone metastatic LoVo cell lines were purchased from Shanghai Fuheng Biotechnology Co., Ltd. An appropriate amount of trypsin was taken, to which an equal volume of complete culture medium was added after most of the cells were removed. The mixture was transferred to a centrifuge tube and centrifuged at 1,000 rpm for 5 min. The supernatant was discarded, and the cell pellet was taken and resuspended in an appropriate amount of complete medium, making a cell suspension. An appropriate amount of cell suspension was added to the culture flask filled with a complete culture medium. All the cells were cultured in a DMEM medium containing 10% fetal bovine serum (FBS) and placed in a humidified incubator at 37°C with 5% CO_2_ concentration.

### 2.3 RNA Isolation and Reverse Transcription Polymerase Chain Reaction (RT-PCR)

The LcVo cells were added to a six-well plate, and each well was digested with TRIzol reagent (Vazyme Nanjing, China). The cell lysate was then pipetted into a Diethy pyrocarbonate (DEPC)-treated EP tube and centrifuged at 12,000 rpm for 15 min at 4°C. The supernatant was taken into a DEPC-treated EP after centrifugation and 0.5 mL isopropanol was added to it. After incubating at room temperature for 10 min, the sample was centrifuged at 12,000 rpm for 10 min at 4°C. The supernatant was discarded, and the pellet was washed with 1.0 mL of 75% ethanol (freshly prepared with DEPC-treated water). Then, the sample was centrifuged at 7,500 rpm and 4°C for 5 min to extract RNA. The reverse transcription reaction was performed using the Vazyme reverse transcription kit (Vazyme Nanjing, China), following the manufacturer’s instructions. The Ct values were identified using the real-time fluorescence detection method. The relative expression level of the *FOXD3* gene was quantified using the 2^-△△Ct^ method with *GAPDH* as expression control. The PCR primers are listed in Table **1**.

### 2.4 siRNA Transfection

The *FOXD3* gene knockdown was performed using siRNA oligonucleotides, which were purchased from Shanghai GenePharma Co., Ltd. The *FOXD3* siRNA sequence was as follows: si-FOXD3 5’-TCATGATGCAGAGCTTCGGCGC TTA-3’. The LoVo cells were cultured in six-well plates at a cell density of 2 × 105 cells/well and grown until they reached the cell confluence of 60-80%. A total of 150 pmol RNA was diluted with reduced serum medium to 200 μL, mixed gently, and left at room temperature for 5 min. Similarly, 6 μL of transfection reagent was diluted to 200 μL with reduced serum medium, mixed gently, and left at room temperature for 5 min. The diluted transfection reagent was added to the diluted RNA dropwise, mixed gently, and left at room temperature for 20 min in order to form RNA complexes. The original culture solution in the six-well plate was aspirated, and the plates were washed twice with reduced serum medium. Then, 1.6 mL of reduced serum medium and 400 μL of RNA complex were added to the six-well plate and gently shaken back and forth to disperse the solutions in the well. The transfected cells were incubated at 37°C for 4-6 h in a CO_2_ incubator. The complexes were then removed, and the medium was replaced. After siRNA transfection for 72 h, the down-regulatory effect of siRNA on *FOXD3* gene expression level was detected using Western blot and real-time PCR.

### 2.5 Cell Migration Assay

The bone-metastatic cells were grown to the logarithmic growth phase in the growth medium and then digested and washed once with PBS. The cells were suspended in a serum-free medium and counted. The cellular concentration was adjusted to 2 × 105 cells/mL. About 600-800 μL of the growth medium, containing 10% FBS was added to a 24-well plate, to which, 100-150 μL of cell suspension was added and incubated for 24 h. The lower surface of the PET film was fixed with 4% paraformaldehyde solution for 30-60 min and stained with crystal violet or trypan blue. The stained PET film was examined microscopically using five visual fields to count the number of cells in the middle and surrounding areas and an average of the cell counts was taken.

### 2.6 CCK8 Assay

The cell suspensions (100 μL/well) were cultured in a 96-well plate for 24, 48, and 72 h in an incubator, having three independent replicates in each group. Then, 10 μL of CCK-8 was added to each well and incubated for 2-4 h. The absorbance was measured at 450 nm using a microplate reader.

### 2.7 Crystal Violet Staining

Each group of cells were digested with 0.25% trypsin and suspended in a 10% serum medium for use. The fold-diluted cells of each group were plated into 12-well plates at 200, 400, and 800 cells per well, respectively. The cells were incubated at 37°C in an incubator having a 5% CO_2_ concentration and saturated humidity for one to two weeks. The culturing was terminated when macroscopic clones appeared in the transparent dish having a flat lid. The supernatant was discarded, and the cells were fixed with 5 mL of 4% paraformaldehyde for 15 min. Then, the fixing solution was removed, and a suitable amount of crystal violet staining solution was added for 10-30 min. After washing off the staining solution with running water and air drying, the colonies with more than 10 cells were counted directly with the naked eye or under a microscope (low power). Finally, the rate of colony formation was calculated.

### 2.8 Western Blot Analysis

The standard protein solution and BCA working solution were prepared using a BCA Protein Assay kit, following the manufacturer’s instructions. The different volumes of standard protein solutions (0, 1, 2, 4, 8, 12, 16, and 20 µL) were added to the wells of the 96-well plate. Then, 200 µL of BCA working solution was added to each well and incubated at 37°C for 20-30 min. The absorbance at a wavelength of 562 nm was measured using a microplate reader. The protein density of each sample was identified from the standard curve and sample volume used. Then, gel electrophoresis was performed and the separated proteins were transferred to the PVDF membrane, which was blocked with 5% skimmed milk powder prepared with TBST for 1 h. Then, the membrane was incubated with the specific primary antibodies, including E-cadherin, N-cadherin, EGFR, K-RAS, P-MEK, and P-MEK, overnight at 4°C to analyze the total expression levels of each protein. After washing off the excess antibodies, the respective secondary antibodies were diluted with TBST appropriately and incubated for 60 min at room temperature. Then, the PVDF membrane was again washed with TBST thrice to remove the excess antibodies and visualized using ECL chemiluminescence.

## 3 RESULTS

### 3.1 FOXD3 was Downregulated in Colon Cancer and Could Interact with Multiple DEGs in Colon Cancer Bone Metastases

**Fig. (1):**
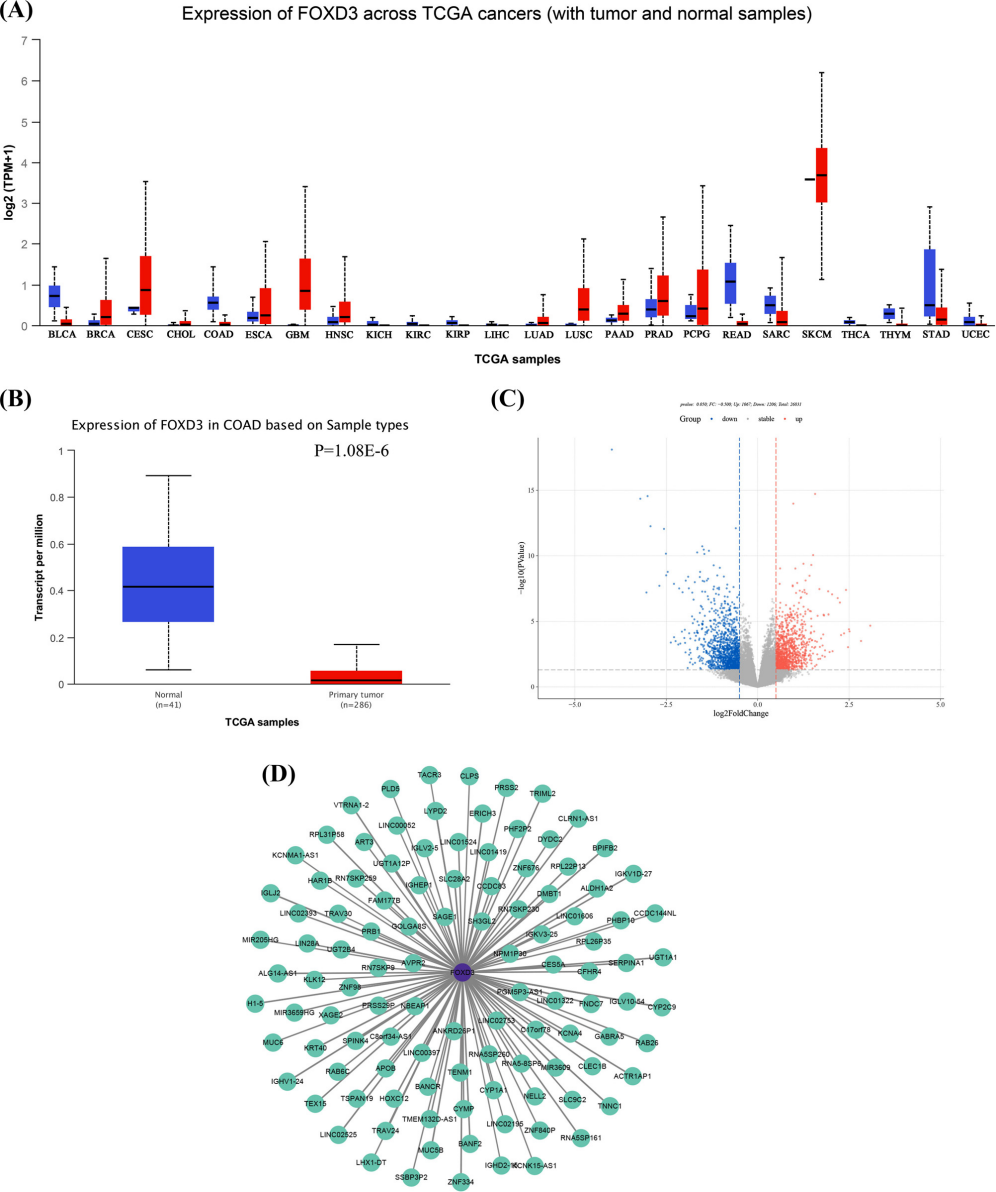
Expression levels of *FOXD3* gene in the colon cancer tissues and its interaction with differentially expressed genes (DEGs) in the colon cancer metastasis. (**A**) Differential expression of *FOXD3* gene in different tumors (data from UALCAN). This figure is freely available on the University of Alabama at Birmingham Cancer Data Analysis Portal (UALCAN) (http://ualcan.path.uab.edu/index.html). (**B**) *FOXD3* expression was high in 41 normal tissues and low in 286 tumor tissues (data from UALCAN). This figure is freely available on the University of Alabama at Birmingham Cancer Data Analysis Portal (UALCAN) (http://ualcan.path.uab.edu/index.html). (**C**) Volcano plot of DEGs in colon cancer and colon cancer metastasis using TCGA database analysis. Red and blue dots represent the upregulated and downregulated genes, respectively (data from TCGA). This figure is freely available on the the TCGA database. (**D**) Network regulation of *FOXD3* and DEGs. The purple and green dots represent *FOXD3* and DEGs (both are positively correlated) (data from TCGA). This figure is freely available on the the TCGA database.

Analyzing the UALCAN public database indicated that the *FOXD3* gene was differentially expressed in various tumor tissues (Fig. **1A**) and its relative expression level in colon cancer (n = 286) tissues was lower than that in the normal tissues (n = 41) (Fig. **1B**). The gene expression data of colon cancer and colon cancer metastasis were obtained from TCGA database and analyzed for the identification of DEGs using the R package DESeq2. A total of 2,273 DEGs, including 1,067 upregulated genes and 1,206 downregulated genes, were identified (Fig. **1C**). The expression profiles of DEGs and the *FOXD3* gene were extracted. The correlation between *FOXD3* and DEDs was identified using Pearson correlation analysis. A total of 395 gene pairs met the set criteria. All the gene pairs were positively correlated after screening the results (Fig. **1D**).

### 3.2 *FOXD3* Gene Knockdown Could Increase the Proliferation of Human Colon Cancer Bone Metastatic Cells

**Fig. (2):**
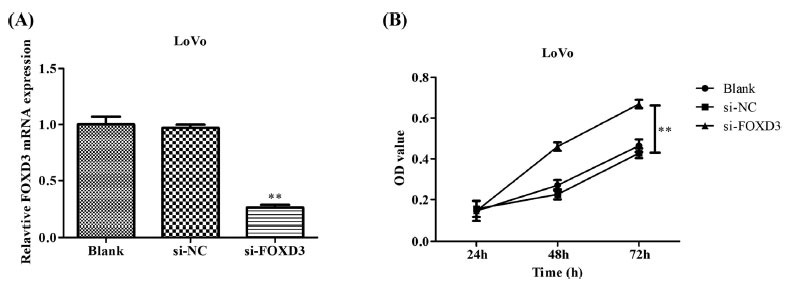
*FOXD3* gene knockdown could increase the proliferation of human colon cancer bone metastatic cells. (**A**) Detection of transfection efficiency in the LoVo cells using Q-PCR. (**B**) CCK8 assay for detecting the effects of *FOXD3* gene knockdown on the cellular proliferation in the LoVo cell line. **P <0.01. **Abbreviations:** si, small interfering; KD, Knockdown; NC, Negative Control; qPCR, Quantitative Polymerase Chain Reaction; OD, Optical Density.

**Fig. (3):**
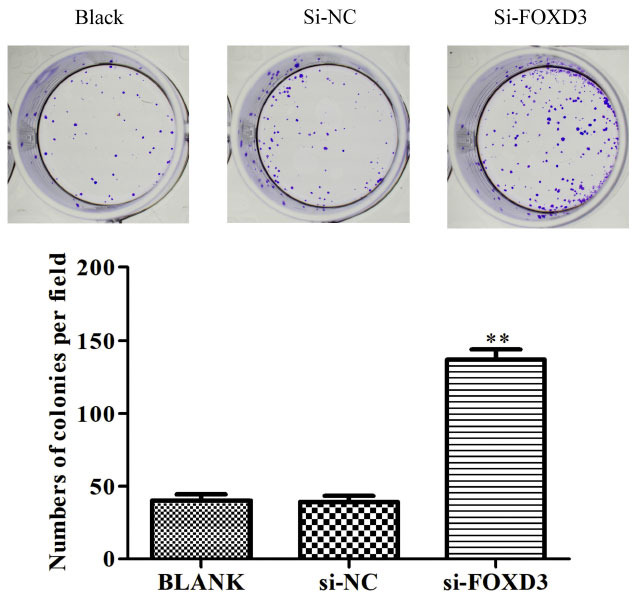
Agar colony formation and statistical analysis of LoVo cells transfected with blank control, siRNA (negative control, NC), and *FOXD3*-siRNA. The experiment was run in three independent replicates. Results represent mean ± SD (n = 9). **P <0.01.

FOXD3 siRNA transfection efficiency was analyzed in the LoVo cell line. The *FOXD3* siRNA plasmid was transfected into the LoVo cells to knock down the expression of the *FOXD3* gene. The Q-PCR analysis revealed that, as compared to those in control (Black) and negative control (Si-NC) cells, the mRNA expression levels of *FOXD3* decreased in these tumor cells in the Si-FOXD3 group (Fig. **2A**). The CCK8 results showed that the knockdown of *FOXD3* expression could significantly promote the proliferation of LoVo cell lines (Fig. **2B**). The growth of LoVo cells in the Si-FOXD3 and control groups was compared using soft agar colony formation assay (Fig. **3**). Consistent with the CCK8 results, the cell proliferation increased significantly in the Si-FOXD3 group as compared to the control group, indicating that *FOXD3* could inhibit the proliferation of colon cancer bone metastatic cells.

### 3.3 *FOXD3* Gene Knockdown Could Increase the Invasive Cell Ability

**Fig. (4):**
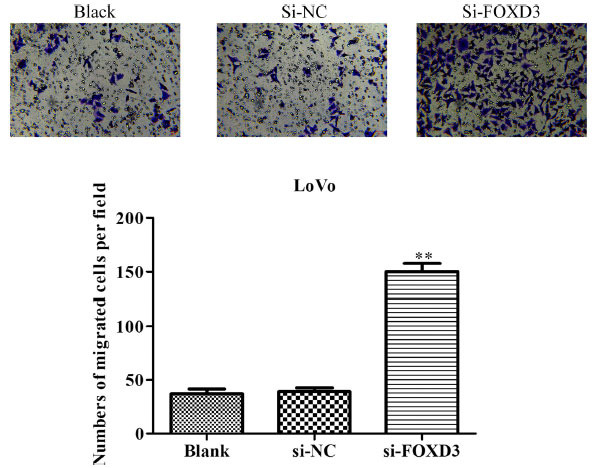
*FOXD3* gene knockdown could increase the metastasis of human colon cancer bone metastatic cells. LoVo cells were transfected with FOXD3 siRNA and negative control for 48 h, and the cells were transferred to an incubator, having 37°C, for 24 h. Then, Transwell cell migration and quantitative assays were performed. **P <0.01.

The *FOXD3* gene knockdown could promote tumor cell proliferation. Therefore, in order to explore the correlations between *FOXD3* and EMT, the role of FOXD3 in regulating the cell invasion ability was investigated using the Transwell cell migration assay. The results showed that, as compared to the control group, the invasive ability of LoVo cells increased significantly after silencing the *FOXD3* gene (Fig. **4**).

### 3.4 *FOXD3* Gene Knockdown Could Activate EGFR/ERK Signaling Pathway and Mesenchymal Attributes in the LoVo Cells

**Fig. (5):**
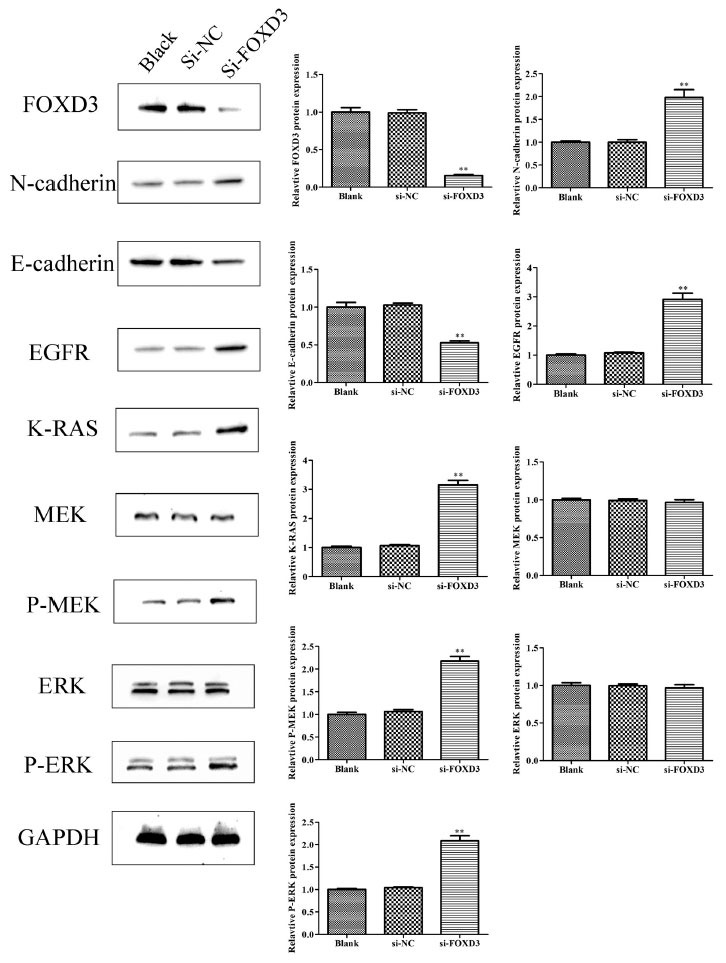
*FOXD3* gene knockdown could activate EGFR/RAS/Raf/MEK/ERK signal pathway in the human colon cancer bone metastatic cells. The blank control (black), negative control (si-NC), and three groups of LoVo cells after FOXD3-siRNA transfection were collected. The proteins were isolated from the LoVo cells. The antibodies for E-cadherin, N-cadherin, EGFR, K-RAS, P-ERK, and P-MEK were used for immunoblot analysis. **P <0.01.

Epithelial-to-mesenchymal transition (EMT) is a pivotal process in cell invasion and tumor progression. This study aimed to discover the effects of EMT epithelial marker E-cadherin and mesenchymal marker N-cadherin expression levels in the si-FOXD3 and control groups using Western blot analysis. The results showed that the *FOXD3* knockdown could result in a significant decrease and increase in the E-cadherin and N-cadherin expression levels, respectively, in the LoVo cells as compared to the control group. *FOXD3* could inhibit the cellular invasion and EMT process in colon cancer bone metastases. Epidermal growth factor receptor (EGFR) and its downstream signaling proteins are involved in the occurrence and development of various human tumors. Furthermore, *FOXD3* expression was upregulated when the B-RAF-MEK-ERK1/2 pathway was inhibited in the B-RAF-mutated melanoma cells. Therefore, this study used Western blot analysis to assess the effect of FOXD3 gene knockdown on the EGFR/ERK signaling pathway. The results showed that the *FOXD3* gene knockdown could increase the levels of EGFR and K-RAS as well as the phosphorylation of MEK and ERK in the LoVo cells (Fig. **5**).

## 4 DISCUSSION

*FOXD3* gene is mainly located near centromeres and telomeres. Recent studies showed that the *FOXD3* gene could interact with other genes through transcription factors and participate in a large number of biological processes, such as cell differentiation, metabolism, proliferation, migration, apoptosis, and tumorigenesis [17, 18]. *FOXD3* gene acts as a tumor suppressor gene in different kinds of tumors. In the present study, the database search showed that the *FOXD3* gene was differentially expressed in various tumor tissues. As compared to normal tissues, the *FOXD3* gene was downregulated in colon cancer tissues, indicating that *FOXD3* might play an inhibitory role in the formation of colon cancer. In addition, comparing colon cancer metastasis and colon cancer cells, numerous DEGs were identified to be associated with the *FOXD3* gene. The *in vitro* experiments showed that the downregulation of the *FOXD3* gene could inhibit the apoptosis of colon cancer bone metastases, enhance the invasion ability of cells, and significantly increase the proliferation of cells. In addition, the downregulation of the *FOXD3* gene in colon cancer bone metastatic cells could also affect the EGFR/ERK signaling pathway and alter the expression levels of EMT-related proteins. Therefore, it was suggested that the *FOXD3* gene could inhibit the occurrence and progression of colon cancer bone metastatic cells by regulating the EGFR/ERK signaling pathway.

Despite the improvements in the treatment of colorectal cancer and prolongation of the patient’s survival time, the incidences of colorectal cancer bone metastasis are continuously increasing. In this study, the results showed that the expression of the *FOXD3* gene was altered in various tumor tissues and downregulated in the colon cancer tissues.

Consistent with these results, numerous previous studies showed that the *FOXD3* gene was downregulated in nasopharyngeal carcinoma [19], lung cancer [20], liver cancer [21], breast cancer [22], and colorectal cancer tissues [23]. In addition, studies also showed that the low expression of *FOXD3* could accelerate the evolution of breast cancer by promoting EMT [13], while its overexpression could significantly inhibit the growth of lung cancer [24], liver cancer [25], and melanoma cells [26]; these results were also consistent with the current study results. Moreover, a study also showed that the *FOXD3* gene could induce tumor cell apoptosis under the endoplasmic reticulum stress through p53 [23], while another study showed that *FOXD3* could inhibit the cellular proliferation of colon cancer cells and activate the EGFR/ERK signaling pathway, thereby acting as a tumor suppressor [27]; these results were also consistent with the current study results. In the current study, in colon cancer bone metastatic cells, the downregulation of the *FOXD3* gene could promote cell growth and migration. Therefore, the *FOXD3* gene might act as a tumor suppressor in the development of human colon cancer bone metastases, thereby indicating its potential as a therapeutic target.

The ERK signaling pathway is essential for intercellular and intracellular communications and regulates basic cellular functions, such as cell growth, survival, and differentiation; the overactivation of this pathway is a driving force in many tumor types [28]. The activating mutations in BRAF oncogene are found in approximately 10% of colorectal cancers [29]. RAS is mutated and activated in about 30% of all the cancer types, among which, the colon cancer mutations account for about 50%, suggesting that the K-Ras mutations are a strong predictor of drug resistance in colorectal cancer metastasis [30]. Yin *et al.* [31] enhanced the MAPK/ERK signaling by silencing the *FOXD3* gene in human ATC cells and showed that *FOXD3* could suppress the occurrence of human thyroid tumors by regulating the MAPK/ERK signaling pathway. Li *et al.* [26] showed that *FOXD3* gene knockout could significantly promote the proliferation of human colon cancer cells and enhance their cell invasion ability. They also showed that the *FOXD3* gene might mediate the occurrence and development of colon cancer through the EGFR/ERK signaling pathway. Studies have shown that the EGFR/ERK signaling pathway might be a new anti-tumor pathway [30]. However, the underlying mechanisms of this signaling pathway and the role of the *FOXD3* gene in colon cancer bone metastasis are still unclear. The current study showed that *FOXD3* gene knockout could significantly activate the EGFR-RAS-Raf-MEK-ERK signaling pathway in human colon cancer bone metastatic cells. These data suggested that the *FOXD3* gene might play a tumor suppressor role by activating the EGFR-RAS-Raf-MEK-ERK signaling pathway, thereby providing a novel target for the treatment of colon cancer bone metastasis, which should be explored in future studies.

## CONCLUSION

In conclusion, this study showed that altered *FOXD3* gene expression could affect the expression levels of EGFR-RAS-Raf-MEK-ERK signaling pathway-related proteins and EMT-related proteins. Moreover, the downregulation of the *FOXD3* gene could also promote the proliferation of LoVo cells, a colon cancer bone metastasis cell line. Therefore, this study suggested that the *FOXD3* gene might inhibit the development of EMT and regulate the proliferation of colon cancer bone metastasis cells through the EGFR-RAS-Raf-MEK-ERK signaling pathway, thereby exhibiting a potential for novel future therapies. There were certain limitations to this study. First, the data obtained from only databases and cell line studies were considered in this study, and the sample data of clinical bone metastases were not available. Second, this study was conducted *in vitro* only. Finally, this study only analyzed the effects of FOXD3 on the proliferation of colon cancer bone metastasis cells and did not further investigate the genes related to colon cancer bone metastasis. Therefore, the mechanism of FOXD3, inhibiting colon cancer bone metastasis, needs to be further explored.

## Figures and Tables

**Table 1:** PCR reaction primers.

**Primer**	**Primer Sequence (5’-3’)**
FOXD3-F	AAGCCCAAGAACAGCCTAGT
FOXD3-R	TGTAGTAGGGGAAGCGGTTG
GAPDH-F	GAAGGTGAAGGTCGGAGTC
GAPDH-R	GAAGATGGTGATGGGATTTC

## Data Availability

The dataset that support the results and findings of this research are available from the corresponding authors [K.W. and Y.C.], upon reasonable request.

## LIST OF ABBREVIATIONS

CRC: Colorectal Cancer
FBS: Fetal Bovine Serum
*FOX*: Forkhead Box
UALCAN: University of Alabama at Birmingham Cancer Data Analysis Portal
logFC: Log Fold-Change
DEGs: Differentially Expressed Genes
EMT: Epithelial-to-Mesenchymal Transition

## References

[1] Siegel R.L., Miller K.D., Jemal A. (2020). Cancer statistics, 2020.. CA Cancer J. Clin..

[2] Siegel R.L., Miller K.D., Fuchs H.E., Jemal A. (2022). Cancer statistics, 2022.. CA Cancer J. Clin..

[3] Xia C., Dong X., Li H., Cao M., Sun D., He S., Yang F., Yan X., Zhang S., Li N., Chen W. (2022). Cancer statistics in China and United States, 2022: Profiles, trends, and determinants.. Chin. Med. J..

[4] Jimi S., Yasui T., Hotokezaka M., Shimada K., Shinagawa Y., Shiozaki H., Tsutsumi N., Takeda S. (2013). Clinical features and prognostic factors of bone metastases from colorectal cancer.. Surg. Today.

[5] Wang J., Li S., Liu Y., Zhang C., Li H., Lai B. (2020). Metastatic patterns and survival outcomes in patients with stage IV colon cancer: A population‐based analysis.. Cancer Med..

[6] Kawamura H., Yamaguchi T., Yano Y., Hozumi T., Takaki Y., Matsumoto H., Nakano D., Takahashi K. (2018). Characteristics and prognostic factors of bone metastasis in patients with colorectal cancer.. Dis. Colon Rectum.

[7] Salim H., Akbar N.S., Zong D., Vaculova A.H., Lewensohn R., Moshfegh A., Viktorsson K., Zhivotovsky B. (2012). miRNA-214 modulates radiotherapy response of non-small cell lung cancer cells through regulation of p38MAPK, apoptosis and senescence.. Br. J. Cancer.

[8] Herman L., Todeschini A.L., Veitia R.A. (2021). Forkhead transcription factors in health and disease.. Trends Genet..

[9] Jin Y., Liang Z., Lou H. (2020). The emerging roles of fox family transcription factors in chromosome replication, organization, and genome stability.. Cells.

[10] Costa R., Muccioli S., Brillo V., Bachmann M., Szabò I., Leanza L. (2021). Mitochondrial dysfunction interferes with neural crest specification through the FoxD3 transcription factor.. Pharmacol. Res..

[11] Zeng Z.L., Zhu H.K., He L.F., Xu X., Xie A., Zheng E.K., Ni J.J., Liu J.T., Zhao G.F. (2020). Highly expressed lncRNA FOXD3-AS1 promotes non-small cell lung cancer progression via regulating miR-127-3p/mediator complex subunit 28 axis.. Eur. Rev. Med. Pharmacol. Sci..

[12] Rosenbaum S.R., Knecht M., Mollaee M., Zhong Z., Erkes D.A., McCue P.A., Chervoneva I., Berger A.C., Lo J.A., Fisher D.E., Gershenwald J.E., Davies M.A., Purwin T.J., Aplin A.E. (2020). FOXD3 regulates VISTA expression in melanoma.. Cell Rep..

[13] Zhao H., Chen D., Wang J., Yin Y., Gao Q., Zhang Y. (2014). Downregulation of the transcription factor, FoxD3, is associated with lymph node metastases in invasive ductal carcinomas of the breast.. Int. J. Clin. Exp. Pathol..

[14] Wu H., Shang J., Zhan W., Liu J., Ning H., Chen N. (2019). miR 425 5p promotes cell proliferation, migration and invasion by directly targeting FOXD3 in hepatocellular carcinoma cells.. Mol. Med. Rep..

[15] Wu Q., Shi M., Meng W., Wang Y., Hui P., Ma J. (2019). Long noncoding RNA FOXD3‐AS1 promotes colon adenocarcinoma progression and functions as a competing endogenous RNA to regulate SIRT1 by sponging miR‐135a‐5p.. J. Cell. Physiol..

[16] Chen Y., Gao H., Li Y. (2020). Inhibition of LncRNA FOXD3-AS1 suppresses the aggressive biological behaviors of thyroid cancer via elevating miR-296-5p and inactivating TGF-β1/Smads signaling pathway.. Mol. Cell. Endocrinol..

[17] Tompers D.M., Foreman R.K., Wang Q., Kumanova M., Labosky P.A. (2005). Foxd3 is required in the trophoblast progenitor cell lineage of the mouse embryo.. Dev. Biol..

[18] Xiao L., Shan Y., Ma L., Dunk C., Yu Y., Wei Y. (2019). Tuning FOXD3 expression dose-dependently balances human embryonic stem cells between pluripotency and meso-endoderm fates.. Biochim. Biophys. Acta Mol. Cell Res..

[19] Xie X., Xiong G., Chen W., Fu H., Li M., Cui X. (2020). FOXD3 inhibits cell proliferation, migration, and invasion in nasopharyngeal carcinoma through regulation of the PI3K–Akt pathway.. Biochem. Cell Biol..

[20] Xu W., Li J., Li L., Hou T., Cai X., Liu T., Yang X., Wei H., Jiang C., Xiao J. (2019). FOXD3 suppresses tumor-initiating features in lung cancer via transcriptional repression of WDR5.. Stem Cells.

[21] He G., Hu S., Zhang D., Wu P., Zhu X., Xin S., Lu G., Ding Y., Liang L. (2015). Hypermethylation of FOXD3 suppresses cell proliferation, invasion and metastasis in hepatocellular carcinoma.. Exp. Mol. Pathol..

[22] Chu T.L., Zhao H.M., Li Y., Chen A.X., Sun X., Ge J. (2014). FoxD3 deficiency promotes breast cancer progression by induction of epithelial–mesenchymal transition.. Biochem. Biophys. Res. Commun..

[23] Xu M., Zhu J., Liu S., Wang C., Shi Q., Kuang Y., Fang X., Hu X. (2020). FOXD3, frequently methylated in colorectal cancer, acts as a tumor suppressor and induces tumor cell apoptosis under ER stress via p53.. Carcinogenesis.

[24] Yan J.H., Zhao C.L., Ding L.B., Zhou X. (2015). FOXD3 suppresses tumor growth and angiogenesis in non-small cell lung cancer.. Biochem. Biophys. Res. Commun..

[25] Liu L.L., Lu S.X., Li M., Li L.Z., Fu J., Hu W., Yang Y.Z., Luo R.Z., Zhang C.Z., Yun J.P. (2014). FoxD3-regulated microRNA-137 suppresses tumour growth and metastasis in human hepatocellular carcinoma by targeting AKT2.. Oncotarget.

[26] Chen X., Gao J., Yu Y., Zhao Z., Pan Y. (2019). LncRNA FOXD3-AS1 promotes proliferation, invasion and migration of cutaneous malignant melanoma via regulating miR-325/MAP3K2.. Biomed. Pharmacother..

[27] Degirmenci U., Wang M., Hu J. (2020). Targeting aberrant RAS/RAF/MEK/ERK signaling for cancer therapy.. Cells.

[28] Li K., Guo Q., Yang J., Chen H., Hu K., Zhao J., Zheng S., Pang X., Zhou S., Dang Y., Li L. (2017). FOXD3 is a tumor suppressor of colon cancer by inhibiting EGFR-Ras-Raf-MEK-ERK signal pathway.. Oncotarget.

[29] Prahallad A., Sun C., Huang S., Di Nicolantonio F., Salazar R., Zecchin D., Beijersbergen R.L., Bardelli A., Bernards R. (2012). Unresponsiveness of colon cancer to BRAF(V600E) inhibition through feedback activation of EGFR.. Nature.

[30] Roberts P.J., Der C.J. (2007). Targeting the Raf-MEK-ERK mitogen-activated protein kinase cascade for the treatment of cancer.. Oncogene.

[31] Yin H., Meng T., Zhou L., Zhao F., Li X., Li Y., Hu M., Chen H., Song D. (2017). FOXD3 regulates anaplastic thyroid cancer progression.. Oncotarget.

